# Camphene, a Plant-Derived Monoterpene, Reduces Plasma Cholesterol and Triglycerides in Hyperlipidemic Rats Independently of HMG-CoA Reductase Activity

**DOI:** 10.1371/journal.pone.0020516

**Published:** 2011-11-03

**Authors:** Ioanna Vallianou, Nikolaos Peroulis, Panayotis Pantazis, Margarita Hadzopoulou-Cladaras

**Affiliations:** 1 Department of Genetics, Development and Molecular Biology, School of Biology, Aristotle University of Thessaloniki, Thessaloniki, Greece; 2 ADNA Inc., Oklahoma City, Oklahoma, United States of America; Griffith University, Australia

## Abstract

**Background:**

Central to the pathology of coronary heart disease is the accumulation of lipids, cholesterol and triglycerides, within the intima of arterial blood vessels. The search for drugs to treat dislipidemia, remains a major pharmaceutical focus. In this study, we evaluated the hypolipidemic properties of the essential oil from Chios mastic gum (MGO).

**Methodology/Principal Findings:**

The hypolipidemic effect of MGO was investigated in naïve as well as in rats susceptible to detergent-induced hyperlipidemia. Serum cholesterol and triglycerides were determined using commercial kits. HMG-CoA (3-hydroxy-3-methylglutaryl coenzyme A) reductase activity was measured in HepG2 cell extracts using a radioactive assay; cellular cholesterol and cholesterol esters were assessed using gas chromatography. MGO administration into naïve rats resulted in a dose-dependent reduction in the constitutive synthesis of serum cholesterol and triglycerides. In hyperlipidemic rats, MGO treatment had also a strong hypolipidemic effect. By testing various components of MGO, we show for the first time that the hypolipidemic action is associated with camphene. Administration of camphene at a dose of 30 µg/gr of body weight in hyperlipidemic rats resulted in a 54.5% reduction of total cholesterol (*p*<0.001), 54% of Low Density Lipoprotein (LDL)-cholesterol (*p*<0.001) and 34.5% of triglycerides (*p*<0.001). Treatment of HepG2 cells with camphene led to a decrease in cellular cholesterol content to the same extend as mevinolin, a known HMG-CoA reductase inhibitor. The hypolipidemic action of camphene is independent of HMG-CoA reductase activity, suggesting that its hypocholesterolemic and hypotriglyceridemic effects are associated with a mechanism of action different than that of statins.

**Conclusions:**

Given the critical role that the control of hyperlipidemia plays in cardiovascular disease, the results of our study provide insights into the use of camphene as an alternative lipid lowering agent and merits further evaluation.

## Introduction

High levels of plasma cholesterol and triglycerides are strongly associated with the development of atherosclerosis and coronary heart disease [1]–[4] and clinical trials designed to reduce serum cholesterol levels by diet or pharmacological means have shown to lead to a reduction in the incidence of this disease [5]. One of the most effective ways of reducing circulating cholesterol levels is to inhibit endogenous cholesterol biosynthesis in the liver as in humans 50% or more of total body cholesterol is derived from de novo synthesis [6]. Further, it has been demonstrated that statins are the predominant inhibitors of HMG-CoA reductase [7] the enzyme that catalyzes the rate determining step of cholesterol biosynthesis. These drugs effectively lower LDL-cholesterol in humans [8] and reduce mortality and morbidity from coronary artery disease [9]–[12]. However, despite the success of treatment by statins there is a need for new therapies to reduce LDL-cholesterol, since some patients do not tolerate statins well and more importantly many patients under statin treatment alone do not achieve the LDL-cholesterol goal according to guidelines suggested by the National Institutes of Health, USA. Recently, several efforts aim to develop more effective hypolipidemic drugs that originate from natural agents.

Chios mastic gum (MG) is a resin produced by the plant *Pistacia lentiscus L.* var *Chia*. MG is produced almost exclusively by plants grown in the southern part of island of Chios in Greece. Repeated attempts have shown that *P. lentiscus* can be cultivated in other parts of the world, but the plant will not produce resin. Since 3000 B.C. Greeks have used MG in diverse applications such as cooking, preparation of beverages, cosmetics, and paints, and treatment of gastric ailments. MG is used to extract mastic gum essential oil (MGO). Since 1997, MG and MGO have been characterized as Products of Protected Origin by the European Union, and rediscovered for their numerous and diverse biomedical and pharmacological properties including, (i) eradication of bacteria and fungi that may cause peptic ulcers, tooth plaque formation and malodor of the mouth and saliva [13]–[24] (ii) amelioration or dramatic reduction of symptoms of auto-immune diseases by inhibiting production of pro-inflammatory substances by activated macrophages, production of cytokines by peripheral blood mononuclear cells in patients with active Crohn's disease, and suppression of production of inflammatory cytokines and chemokines in a mouse asthma model [25]–[28]; (iii) anti-inflammatory and anti-oxidant properties [25]–[31]; (iv) protection of the cardiovascular system by effectively lowering the levels of serum cholesterol and protection of LDL from oxidation [30], [31]; (v) antitumor growth activities against several cancer types (leukemia, prostate, colon, lung and melanoma cancer) [32]–[40] and (vi) improvement of symptoms in patients with functional dyspepsia [41].

MG and MGO are highly insoluble in water, but somewhat soluble in different organic solvents. Their chemical composition has been analyzed, and several constituents have been isolated and identified in various fractions [15], [17], [20], [42]–[45]. In general, of the 69 MGO constituents, 61 have been identified. Six constituents β-caryophyllene, α-pinene, β-pinene, camphene, β-myrcene, and linalool comprise 65% to 80% of the weight of all product extracted. The same major constituents were found in both MG and MGO, but MG proved to be more difficult to handle than MGO [17]. In this report, we present experimental evidence that MGO can prevent hyperlipidemia, that is, prevent increase of total cholesterol, LDL-cholesterol and triglycerides following their induction in a rat model. The effects of various MGO components on the content of plasma lipids in hyperlipidemic rats were also investigated in order to determine the active hypolipidemic component. The hypolipidemic activity of MGO was unequivocally associated with camphene and it was also demonstrated that the lipid-lowering effect of this monoterpene proves to be dose-dependent. We also report that the hypolipidemic activity of camphene in hepatic cells is mediated via a metabolic process distinctly different than the process of inhibition of HMG-CoA reductase induced by statins. This study also compares the efficacy of camphene in lowering intracellular cholesterol and cholesterol ester levels with that of mevinolin, a potent lipid lowering drug. Treatment with camphene exhibited no cytotoxicity in human hepatic cells. Thus, camphene may develop as an alternative lipid lowering agent, whereas further investigation is warranted in order to elucidate its molecular mechanism of action.

## Methods

### Materials

MGO was obtained by steam distillation and was kindly donated by the Association of Chios Mastic Gum Producers (Chios, Greece). The MGO constituents, α-pinene, β-pinene, myrcene, linalool, β-caryophyllene and camphene, mevinolin, the detergents, Triton WR-1339 and Tween-80, and the MTT (3-(4,5-Dimethylthiazol-2-yl)-2,5-diphenyltetrazolium bromide) were from Sigma-Aldrich (St. Louis, MO). Triton WR-1339 was used for induction of hyperlipidemia, and Tween-80 as emulsifier. [^14^C]HMG-CoA (10 µCi), [2-^14^C] acetic acid sodium salt (250 µCi) and [1-^14^C] oleic acid (50 µCi) were from Amersham Biosciences (UK Ltd). Resin AG 1-X8 formate-form and reagents for protein determination were obtained from BioRad (Berkeley, CA, USA). The enzymatic determination of total cholesterol, LDL-cholesterol and triglycerides in plasma were assayed using commercially available kits obtained from Randox laboratories Ltd (Antrim, UK).Reagents for protein determination were from Bio-Rad. The human hepatic cell line, HepG2, was purchased from ATCC (Manassas, VA, USA). Cell culture medium, Dulbecco's modified Eagle's medium (DMEM), fetal bovine serum (FBS), and antibiotics, penicillin and streptomycin, were from Gibco BRL Life Technologies (Gaithersburg, MD, USA). Lipoprotein-deficient serum (LPDS) was obtained from Autogen Bioclear UK Ltd (Wilts, UK).

### Ethics statement

Animals received proper care in compliance with the “Guidelines for the Care and Use of Laboratory Animals” published by the Greek Government (160/1991) based on EU regulations (86/609) All experiments were reviewed and approved by the Institutional Animal Care and Use Committee at Aristotle University of Thessaloniki (protocol #13/16588).

### Animal experimentation

Female Fisher-344 rats, 2–4 months old and weighing 180–200 g each were used in this study. The animals were housed under standard laboratory conditions (18°C–20°C, 12 h light and dark cycle) and received a diet of commercial food pellets and tap water *ad libitum*. Triton WR-1339 was stirred in KCl 1.15% until dissolved and doses of 200 mg/kg were injected intraperitoneally (ip) during about 25 sec with the aid of ether anaesthesia. MGO and MGO components were administered intraperitoneally (ip) into the rats at the desired doses (depending on the study) in 1 ml of a carrier consisting of 10% Tween-80 (v/v) in normal saline. Control animals received 1 ml of carrier alone (placebo). Triton WR-1339 was administered ip to rats 1 h after ip administration of the tested compound dissolved in carrier. Rats were deprived of food prior to Triton WR-1339 administration, but they were allowed free access to drinking water. Each animal group (for each compound treatment) consisted of six rats. For studies in hyperlipidemic rats, one group received 1 ml carrier and served as control #1 (placebo); the second group of animals received Triton alone and served as control #2 for hyperlipidemia induction; and the third animal group received treatment with various MGO constituents in 1 ml carrier one hour prior to administration of Triton WR-1339. The hypolipidemic activity of MGO and its constituents was concluded by comparing the lipid contents in plasma from animals treated with Triton alone and in plasma from animals treated with Triton and MGO or MGO constituents. Twenty-four hours later, blood was collected from the hearts of animals in heparinised tubes, centrifuged to obtain plasma and stored at −80°C. The levels of total cholesterol, LDL-cholesterol, and triglycerides in plasma were measured using commercially available diagnostic kits according to instructions provided by the manufacturer.

### Cell culture

HepG2 cells were cultured in DMEM supplemented with 10% FBS, penicillin (100 units/ml) and streptomycin (0.25 µg/ml) at 37°C in a humidified atmosphere of 5% CO_2_. The cell cultures were routinely passaged, while growing exponentially. The test compounds were dissolved in absolute ethanol prior to appropriate dilution with cell culture medium so that the final ethanol concentration never exceeded 0.5% (vol/vol) in the culture. Cell viability and protein content was determined by the trypan blue dye method and by the Bio-Rad protein assay, respectively.

### Cell treatment with various compounds and preparation of cell extracts

On day 0, 3×10^5^ cells were seeded in cell culture dishes (60-mm diameter) in DMEM containing 10% FBS. On day 3 or 4, the medium was replaced with FBS-containing fresh medium, which was subsequently replaced on day 6 with DMEM containing 10% LPDS, instead of FBS, followed by incubation for 24 h. On day 7, the cells were pre-incubated for 18 h with camphene and mevinolin in fresh DMEM containing LPDS. Following incubation, the cells were washed twice with DMEM and incubated in DMEM for another 15 min to remove intracellular material. Subsequently, the cells were washed three times with cold PBS, pH 7.4, and detached from the substrate with the aid of a rubber policeman. PBS containing the detached cells was centrifuged at 2000×g for 5 min at 4°C, the clarified supernatant was discarded, and the cell pellet was stored at −80°C until used.

Cell extracts were prepared according to a published procedure [46]. Briefly, the cell pellet was suspended in 100 µl of a lysis buffer containing 50 mM imidazole, pH 7.4, 250 mM NaCl, 2 mM EGTA, 1 mM EDTA, 5 mM dithiothreitol, 50 µM leupeptine and 0.25% Brij 97. After incubation for 30 min at 37°C the homogenate was centrifuged for 10 min at 12000×g at 4°C and aliquots of the supernatant were used to assay for HMG-CoA reductase activity. The cellular protein content was determined using the Bio-Rad protein assay.

### MTT assay

Cell viability of HepG2 cells was evaluated using the MTT assay [47]. Briefly, cells were seeded into 96-well plate with an initial density of 1×10^5^ cells per well. After 24 h of incubation various concentrations of camphene were added. Each treatment was performed in triplicates. Cells were incubated for another 24 h in a 5% CO_2_ incubator at 37°C. Four hours prior to termination of the incubation, medium was discarded and 100 µl of MTT (1 mg/ml in PBS) were added to each well, to allow the formation of formazan crystals. Then the medium was discarded, 100 µl/well of DMSO was added to dissolve the formazan crystals, and the plates were incubated for 10 min at room temperature. The optical absorbance value at 540 nm was detected with a microplate reader. The absorbance value that was determined for cells cultured in complete media without camphene corresponded to 100% viability.

### HMG-CoA reductase activity assay

Twenty-µl of the cell extract (see above) were vigorously mixed with 30 µl of cell lysis buffer and then incubated at 37°C for 15 min. The reaction was initiated by the addition of 25 µl of a solution containing 50 mM imidazole, pH 7.4, 250 mM NaCl, 2 mM EGTA, 76 mM EDTA, 5 mM DTT, 90 mM glucose-6-phosphate, 0.75 units of glucose-6-phosphate dehydrogenase, 2.5 mM NADP^+^ and 30 µM [^14^C]HMG-CoA (0.02 µCi). Controls consisted of 50 µl lysis buffer alone. The samples were incubated for 60 min at 37°C and 25 µl of 5 M HCl were added to terminate the reaction. After standing for 30 min at 37°C to allow for lactonization of the mevalonic acid the samples were centrifuged for 3 min at 10000×g to pellet denatured protein. [^14^C] mevalonolactone was separated from [^14^C]HMG-CoA on a small column packed with ion exchange resin (AG 1-X8 formate-form). The column was made up in water and the sample was eluted 3× with 1 ml of water. The eluates were collected and radioactivity was measured in a liquid scintillation counter (Quicksafe A, Zinsser Analytic). Enzyme activity was expressed in counts per minute.

### Determination of cell cholesterol

On day 7 (see: Treatment of HepG2 cells), the cells were pre-incubated for 1 h and 18 h with camphene and mevinolin in DMEM containing 10% LPDS. Following incubation the cells were washed 3× with cold PBS, detached with the aid of a rubber policeman and collected in 1 ml of PBS. The protein content was determined and lipids were extracted by the method of Bligh and Dyer [48]. Briefly, appropriate volumes of chloroform and methanol were added to cells suspended in 1 ml of PBS in order to obtain a one-phase system consisting of CHCl_3_/CH_3_OH/H_2_O (1∶2∶0.8, v/v/v). After vigorous mixing, the appropriate volumes of chloroform and water were added to obtain a two-phase system CHCl_3_/CH_3_OH/H_2_O at 1∶1∶0.9 ratios (v/v/v) followed by another vigorous mixing. Then, the mixture was allowed to equilibrate for 30 min at −20°C until the two phases were separated. The chloroform phase containing total lipids was removed and evaporated under nitrogen stream, then the lipids were dissolved in CHCl_3_/CH_3_OH (2∶1, v/v), and cholesterol was determined by gas chromatography (Hewlett Packard 5890 Series II). One-hundred µg of cholestane in chloroform was added, as an internal standard, to a volume of cell suspension containing 1 mg of protein. The temperature program was started at 180°C, increased to 300°C at a rate of 15°C/min and maintained at 300°C for 12 min.

### Cholesterol ester determination

Lipids were extracted by the method of Bligh and Dyer [48]. Cholesterol ester in chloroform was added as an internal standard. The lipids were dissolved in a mixture of CHCl_3_/CH_3_OH (2∶1, v/v) and separated by Thin Layer Chromatography (TLC) using hexane/diethylether/acetic acid as the developing system. Lipid spots were located under ultraviolet light after spraying with a solution of dichlorofluorescein in ethanol, and each spot was excised and transmethylated in methanolic sodium methoxide followed by treatment with methanolic boron trifluoride. The cholesterol esters thus produced were extracted with hexane and separated in a gas chromatograph. The temperature program started at 160°C and increased to 270°C at a rate of 5°C/min.

### Statistical analysis

Mean and standard error of means are reported. Significant differences between values of treated and control groups were determined using the unpaired Student's *t* test (two-tailed) or the Student-Newmann-Keuls test.

## Results

### Effect of MGO on plasma lipid levels in naïve animals

We initially determined whether MGO could affect the lipid contents in the plasma of naïve rats prior to induction of hyperlipidemia. For this, we treated naïve animals with two different MGO doses, 2.5% and 4%, and then compared the lipid contents in the plasma before and after MGO administration. After treatment with 2.5% MGO there were decreases in total cholesterol, LDL-cholesterol, and triglycerides by 29%, 23.9%, and 21.2%, respectively, whereas, the 4% MGO dose resulted in reductions of 53.6%, 45.2% and 30.6% in the contents of total cholesterol, LDL-cholesterol and triglycerides, respectively (Table 1). These results indicated that the 4% MGO dose was more effective than the 2.5% dose in reducing the lipid contents in the plasma. Therefore, we assumed that the extent of lipid reduction could be dependent on the MGO dose administered into the animals. Furthermore, MGO administration did not affect food intake.

**Table 1:** Effect of various MGO concentrations on contents of total cholesterol, LDL-cholesterol and triglycerides in the plasma of naïve rats (without induction of hyperlipidemia).

		Rat group		
MGO dose	Plasma lipid content	Placebo	+MGO	Lipid content decrease
				after MGO treatment (%)
2.5%	Total cholesterol	57.3±4.4	40.7±4.2 (<0.0001)	29.0
2.5%	LDL cholesterol	18.5±1.6	14.0±1.0 (0.0002)	23.9
2.5%	Triglycerides	85.5±15.0	67.5±15.7 (0.07)	21.1
4%	Total cholesterol	69.3±8.5	32.1±6.6 (<0.0001)	53.6
4%	LDL cholesterol	13.2±2.2	7.3±1.4 (<0.0003)	45.2
4%	Triglycerides	114.8±14.4	79.6±14.20 (0017)	30.6

The placebo (control) group was administered 1 ml carrier alone. The MGO treated group received a dose of MGO cocktail. The injected cocktails of different doses of MGO consisted of: 2.5% or 4% MGO (v/v) in 1 ml carrier. Twenty-four h later, blood was collected from the hearts of animals in heparinized tubes and plasma samples were prepared and assayed for total cholesterol, LDL-cholesterol, and triglycerides. The values represent mean ± SD of six rats. *p*-values of unpaired Student's t test (two-tailed) are shown in parentheses (MGO-treated rat groups vs placebo-receiving group).

### Hypolipidemic activity of MGO on plasma lipids in hyperlipidemic rats

The effect of MGO on plasma cholesterol and triglyceride levels was assessed in a rat model in which the circulating levels of total cholesterol, LDL-cholesterol and triglycerides were substantially increased following administration of the detergent, Triton WR-1339. This compound has been widely used for induction of acute hyperlipidemia in several animal models [49]–[51] and for screening natural and chemical hypolipidemic drugs [52], [53]. The rationale of the experiments with hyperlipidemic rats is described in the methods section. MGO was administered at doses of 2.5%, 4%, 5% and 7.5% (v/v). As seen in Table 2, column A: placebo, the content of each plasma lipid category was similar in all control (placebo) animals, ranging from about 53 to 61 mg/dl for total cholesterol, 9 to 10 mg/dl for LDL-cholesterol, and 140–144 mg/dl for triglycerides. Administration of Triton WR-1339 caused a dramatic increase in the content of plasma lipids ranging from 416 to 628 mg/dl for total cholesterol, 119 to 270 mg/dl for LDL-cholesterol, and 2752 to 4673 mg/dl for triglycerides (Table 2, column B: Triton WR-1339). However, the induction in lipid content was lower in animals which had received various MGO doses prior to Triton administration. Thus, in hyperlipidemic animals treated with various MGO doses, the plasma contents were 168 to 375 mg/dl for total cholesterol, 38 to 130 mg/dl for LDL-cholesterol, and 1088 to 2571 mg/dl for triglycerides, respectively (Table 2, column C: MGO+Triton WR-1339). Table 2 shows that plasma lipid contents were not significantly different between hyperlipidemic animals and hyperlipidemic animals that had received a 2.5% MGO (*p*>0.05). In contrast, significant decreases were observed in total cholesterol (55.8%), LDL-cholesterol (51.8%) and triglycerides (50.1%) in hyperlipidemic animals receiving a 4% MGO dose with *p*<0.001. Moreover, rats that received a 5% or 7.5% MGO dose exhibited further reductions in the various lipid contents with *p*<0.001 (Table 2). Thus, it appears that the most effective inhibition occurred when a 4% MGO dose was administered in the rats, and that MGO doses above 4% resulted in a small additional or no further inhibition of lipid contents. Taken these results collectively we concluded that 4% was the optimal MGO dose, since it was the lowest one that decreased plasma lipids to more than 50 percent.

**Table 2:** Effect of various MGO concentrations on contents of total cholesterol, LDL- cholesterol and triglycerides in the plasma of Triton WR-1339 treated rats.

		Rat Group			
MGO	Plasma lipid	(A)	(B)	(C)	Percentage of lipid
dose	content(mg/dl)	Placebo	+Triton WR-1339	+MGO	decrease in rat group (C)
				+Triton WR-1339	compared to rat group (B)
2.5%	Total cholesterol	57.3±7.0	416.0±53.0	375.4±31.4^ns^	9.75
2.5%	LDL-cholesterol	9.2±2.0	119.2±22.9	108.5±16.1^ns^	9.0
2.5%	Triglycerides	143.5±32.8	2752.4±580.7	2571.4±469.4^ns^	6.6
4%	Total cholesterol	60.5±9.3	483.5±37.6	213.5±18.5^***^	55.8
4%	LDL-cholesterol	10.3±1.9	270.6±23.9	130.3±21.1^***^	51.8
4%	Triglycerides	142.0±28.5	4673.3±400.7	2331.3±252.4^***^	50.1
5%	Total cholesterol	52.8±3.5	628.4±32.1	256.5±29.0^***^	59.2
5%	LDL-cholesterol	10.2±0.9	219.6±22.9	64.5±9.0^***^	70.6
5%	Triglycerides	39.8±10.4	4545±54.2	2043.2±102.0^***^	55.0
7.5%	Total cholesterol	58.6±7.6	420.6±27.8	168.6±20.5^***^	59.9
7.5%	LDL-cholesterol	10.1±2.8	138.4±8.6	38.0±3.7^***^	72.5
7.5%	Triglycerides	139.8±28.0	3146.9±294.4	1088.2±144.5^***^	65.4

The placebo (control) group (A) was administered 1 ml carrier alone. The Triton WR-1339 group (B) was administered 1 ml carrier and one hour later 1 ml of Triton WR-1339 (200 mg/kg). The MGO-treated group (C) received a dose of MGO and one h later a single dose of Triton WR-1339. MGO was administered at 2.5%, 4%, 5% and 7.5% (v/v) in 1 ml carrier. Controls and MGO were injected intraperitoneally. Twenty-four h later, blood was collected from the hearts of animals in heparinized tubes and plasma samples were prepared and assayed for total cholesterol, LDL-cholesterol, and triglycerides. The values represent mean ± SD of six rats. Significantly different values were obtained from MGO-treated rats as compared to Triton WR-1339 treated rats with *p*<0.001 (***) by the Student-Newmann-Keuls test; ns, non significant (*p*>0.05).

### Effect of various MGO constituents on plasma lipids in hyperlipidemic rats

As mentioned in the Introduction section the chemical composition of MG, MGO and resins extracted from the insect galls on *P. lentiscus* have been analyzed, and several constituents have been isolated and identified in various fractions. More than 80% of MGO consists of 66.48% α-pinene, 3.29% β-pinene, 8.34% myrcene, 2.84% linalool, 2.04% β-caryophyllene and 0.83% camphene [42]. To examine the effect of major MGO constituents, each constituent was administered separately to rats at a dose identical to that contained in the optimal MGO dose (4%). No significant changes were measured in the plasma lipids of hyperlipidemic animals treated with α-pinene, β-pinene, myrcene, linalool and β-caryophyllene, whereas, a significant reduction in lipids was observed in the plasma of the animals treated with camphene (*p*<0.001; Table 3). Thus, in the camphene treated rats the total cholesterol content was decreased 19.4%, LDL-cholesterol by 18.1% and triglycerides by 22.9% (Table 3). These results indicated that the lipid-lowering activity of MGO was associated with one of its components namely camphene.

**Table 3:** Effect of various MGO components on plasma lipid contents in hyperlipidemic rats.

		Rat Group			
MGO	Plasma lipid	(A)	(B)	(C)	Percent
component	content	Placebo	+Triton WR-1339	+MGO component	change
	(mg/dl)			+Triton WR-1339	
α-pinene	Total cholesterol	69.0±6.0	354.0±27.7	350.6±38.1^ns^	−1.0
α-pinene	LDL-cholesterol	13.6±2.7	130.6±13.6	134.0±17.6^ns^	+2.6
α-pinene	Triglycerides	130.4±20.8	2067.6±295.3	2082.7±376.2^ns^	+0.7
β-pinene	Total cholesterol	69.0±6.0	354.0±27.7	346.1±21.0^ns^	−2.3
β-pinene	LDL-cholesterol	13.6±2.7	130.6±13.6	130.5±16.2^ns^	−0.1
β-pinene	Triglycerides	130.4±20.8	2067.6±295.3	1930.7±252.8^ns^	−6.6
myrcene	Total cholesterol	69.5±5.1	335.0±25.4	333.6±33.5^ns^	−0.4
myrcene	LDL-cholesterol	12.8±2.3	97.1±16.4	95.9±17.2^ns^	−2.1
myrcene	Triglycerides	132.5±11.0	2186.4±363.1	2147.9±398.5^ns^	−1.7
β-caryophyllene	Total cholesterol	69.5±5.1	335.0±25.4	329.6±24.9^ns^	−1.6
β-caryophyllene	LDL-cholesterol	12.8±2.3	97.1±16.4	97.1±12.9^ns^	−0.3
β-caryophyllene	Triglycerides	132.5±11.0	2186.4±363.1	2078.5±353.7^ns^	−4.9
linalool	Total cholesterol	69.5±5.1	335.0±25.4	339.4±29.3^ns^	+1.3
linalool	LDL-cholesterol	12.8±2.3	97.1±16.4	102.4±13.0^ns^	+6.1
linalool	Triglycerides	132.5±11.0	2186.4±363.1	2184.8±384.7^ns^	−0.1
camphene	Total cholesterol	68.3±12.9	280.4±21.5	226.0±18.5^***^	−19.4
camphene	LDL-cholesterol	12.9±3.4	49.4±6.1	36.4±6.8^**^	−26.3
camphene	Triglycerides	124.5±28.0	1920.5±197.8	1481.5±219.5^***^	−22.9

The placebo group (A) was administered 1 ml carrier alone, whereas, the Triton WR-1339 group (B) was administered 1 ml carrier and one h later 1 ml of Triton WR-1339 (200 mg/kg of body weight). The rats in group (C) were treated with various MGO components and one h later were treated with Triton WR-1339. Each MGO-component was administered at a dose identical to that contained in the 4% dose of MGO. Twenty-four h later, blood was collected from the hearts of animals in heparinized tubes, and plasma samples were prepared and assayed for total cholesterol, LDL-cholesterol, and triglycerides. The percent change caused by the various MGO components (C) is expressed relative to the group of Triton WR-1339-treated rats (B), which was taken as 100%. The values represent mean ± SD of six rats. Values of camphene treated rats were significantly different as compared to Triton WR-1339 treated rats with *p*<0.01 (**) and *p*<0.001 (***) by the Student-Newmann-Keuls test. Values of α-pinene, β-pinene, myrcene, β-caryophyllene and linalool treated rats vs Triton WR-1339 treated rats were non significant, ns (*p*>0.05).

### The hypolipidemic effect of MGO is unequivocally associated with camphene

To further assure that the hypolipidemic activity of MGO is linked to the presence of camphene, we carried out an experiment in which the rats were administered two mixtures, A and B, of MGO constituents. Mixture A consisted of α-pinene, β-pinene, myrcene, linalool, and β-caryophyllene, and Mixture B consisted of Mixture A+camphene. Mixture A and Mixture B were prepared so that the dose of each constituent was equal to that contained in 4% MGO. As it is shown in Table 4, treatment with Mixture A (without camphene) had no significant effect on plasma lipids (*p*>0.05), whereas, rats treated with Mixture B (containing camphene) resulted in a significant decrease in plasma lipids. Specifically, administration of mixture B to hyperlipidemic rats resulted in total cholesterol reduction by 40.4%, LDL-cholesterol reduction by 61.1%, and triglyceride reduction by 54.4%, when compared to hyperlipidemic rats treated with Triton WR-1339 alone (*p*<0.001). These results indicated that camphene was indeed the MGO constituent exhibiting the hypolipidemic activity.

**Table 4:** The anti-hyperlipidemic action of MGO is associated with camphene.

	Rat Group				Percent change	
Plasma lipid	(A)	(B)	(C)	(D)	+Mixture	
content (mg/dl)	Placebo	Triton WR-1339	+Mixture A	+Mixture B	A	B
			+Triton WR-1339	+Triton WR-1339		
Total cholesterol	65.1±6.9	262.9±32.3	263.4±23.7^ns^	156.7±12.3^***^	+1.2	−40.4
LDL-cholesterol	16.4±2.8	47.6±9.1	45.4±10.7^ns^	18.5±2.7^***^	−4.7	−61.1
Triglycerides	130.2±22.6	1950.8±191.1	1976±140.7^ns^	888.7±99.3^***^	+1.3	−54.4

The placebo group (A) was administered 1 ml carrier alone. The Triton WR-1339 group (B) was administered 1 ml carrier and one h later 1 ml of Triton WR-1339 (200 mg/kg). Animals in (C) and (D) received Mixture A and Mixture B, respectively, in 1 ml carrier and one hour later received Triton WR-1339. Mixture A consisted of α-pinene, β-pinene, myrcene, β-caryophyllene and linalool; and Mixture B consisted of Mixture A+camphene. The constituents in Mixtures A and B were present at concentrations identical to those contained in the 4% MGO dose. The percent change caused by Mixture A and B treatments is expressed relative to measurement in the group of Triton WR-1339-treated rats (B), which was defined as 100%. The values represent mean ± SD of six rats. Significantly different values were obtained in Mixture B-treated rats compared to Triton WR-1339-treated rats with *p*<0.001 (***) by the Student-Newmann-Keuls test. Values of Mixture A-treated rats vs Triton WR-1339-treated rats were non significant, ns (*p*>0.05).

### Lipid-lowering effects of various camphene concentrations in hyperlipidemic rats

After determining that camphene was the MGO constituent exhibiting anti-hyperlipidemic activity, we investigated whether this activity was dependent on the camphene concentration. For this study, various camphene doses were injected into rats one hour prior to injection of the hyperlipidemia-inducing agent, Triton WR-1339. The camphene doses were 1.5, 7.5 and 30 µg/g of body weight. Control animals received Triton WR-1339 alone. As previously observed, injection of Triton WR-1339 resulted in dramatic increases in total cholesterol, LDL-cholesterol and triglycerides as shown in Table 5 (compare column B to column A). However, the Triton-induced increase in lipid content was attenuated in the rats that had received camphene prior to Triton injection (compare column C to column B). Further, the extent of attenuation of the lipid content increase was positively correlated with the camphene dose. Thus, the percentage of attenuation for each lipid category was minimal in rats injected with a 1.5 µg/g dose, and maximal in rats injected with a 30 µg/g dose (Table 5). Further, the 30 µg/g dose resulted in 50% or higher attenuation in the contents of total cholesterol (54.5%) and LDL-cholesterol (54.0%). Also, the 30 µg/g dose resulted in a dramatic attenuation of Triton-induced increase in triglycerides content (34.5%). It is apparent that a camphene dose of 7.5 µg/g body weight was the lowest dose used to accomplish the maximal inhibition in LDL-cholesterol and triglyceride contents. However, camphene doses higher than 7.5 µg/g were required to accomplish extensive inhibition of total cholesterol content. A possible explanation is that total cholesterol consists of lipid forms, in addition to LDL-cholesterol, which are not affected by camphene. It should be noted that this higher camphene dose (30 µg/g) corresponds to a 20-fold higher dose of camphene that is present at the 4% MGO dose. Therefore, the anti-hyperlipidemic effect of camphene appears to be dose-dependent with more than 50% of its effectiveness exhibited when a 7.5 µg/g dose was used. Finally, no toxicity was observed in the animals even after receiving the highest camphene dose of 30 µg/g (or 30 mg/kg) of body weight. Apparently, this dose was well-tolerated and it was much lower than the reported camphene LD_50_ for rats which was shown to be higher than 5 g/kg of body weight (CAS No. 79-92-5: Screening information Data Set (SIDS) of OECD High Volume Chemicals Programme, 1993).

**Table 5:** Effect of various camphene concentrations on contents of total cholesterol, LDL- cholesterol and triglycerides in the plasma of Triton WR-1339-treated rats.

		Rat Group			
Camphene	Plasma lipid	(A)	(B)	(C)	Lipid decrease
dose per	content (mg/dl)	Placebo	+Triton WR-1339	+camphene	in rat group (C)
body weight				+Triton WR-1339	compared to
(µg/g)					rat group (B) (%)
1.5	Total cholesterol	68.3±12.9	280.4±21.5	226.0±18.5^***^	19.4
1.5	LDL-cholesterol	12.9±3.4	49.4±6.1	36.4±6.8^**^	26.3
1.5	Triglycerides	124.5±28.0	1920.5±197.8	1481.5±219.5^***^	22.9
7.5	Total cholesterol	70.3±6.2	342.1±27.6	231.2±16.1^***^	32.4
7.5	LDL-cholesterol	14.2±2.8	99.2±11.8	53.7±7.1^***^	45.9
7.5	Triglycerides	136.4±13.9	2875.0±180.7	2042.3±153.4^***^	29.0
30	Total cholesterol	72.0±9.1	529.1±85.8	240.8±22.5^***^	54.5
30	LDL-cholesterol	13.2±3.1	284.5±39.9	131.1±9.4^***^	54.0
30	Triglycerides	120.5±15.5	2443.6±126.0	1600.3±20.3^***^	34.5

The placebo group (A) was administered 1 ml carrier alone, whereas the Triton WR-1339 group (B) was administered 1 ml carrier and one h later 1 ml of Triton WR-1339 (200 mg/kg). The camphene treated group (C) received a dose of camphene and one h later a single dose of Triton WR-1339. Camphene was administered at 1.5, 7.5 and 30 µg/g of body weight. Controls and camphene were injected ip. Twenty-four hours later, blood was collected from the hearts of animals in heparinized tubes, and plasma samples were prepared and assayed for total cholesterol, LDL-cholesterol, and triglycerides. The values represent mean ± SD of six rats. Significantly different lipid contents were determined in the camphene treated rats (C) compared to rats treated with Triton WR-1339 alone (B). *p*<0.01 (**), and *p*<0.001 (***) by the Student-Newmann-Keuls test.

### Effect of camphene on intracellular cholesterol content

**Figure 1:**
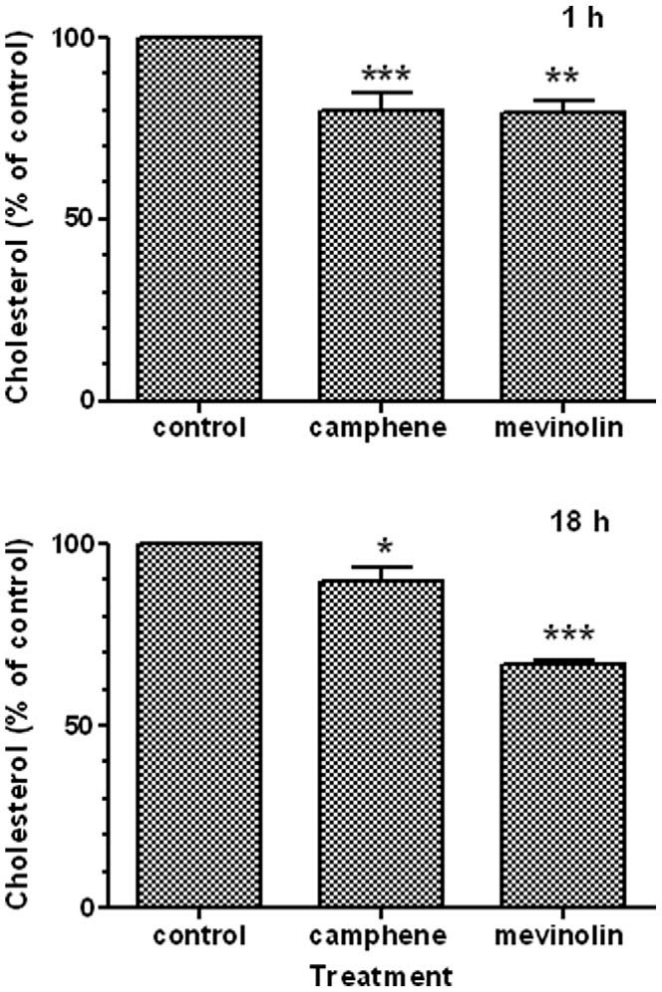
Effect of camphene and mevinolin on cholesterol levels in HepG2 cells. On day 7 (see Methods), HepG2 cells were incubated with camphene (37 µM) and mevinolin (37 µM) for 1 h and 18 h. Cholesterol was extracted and quantified by gas chromatography. The cholesterol concentration in treated cells was plotted relative to the concentration in untreated control cells (100%). Results are from three independent experiments in triplicates and expressed as the mean ± SD. *p*<0.05 (*); *p*<0.01 (**); and *p*<0.001 (***) by the Student-Newmann-Keuls test.

The *in vivo* studies demonstrated the hypolipidemic activity of camphene. It is well known that the commonly used drugs, statins, lower hyperlipidemia by inhibiting HMG-CoA reductase, the cellular enzyme required for cholesterol biosynthesis [8]. To study the effect of camphene on cholesterol biosynthesis, the HepG2 cell line was used as a model. This cell line has been widely used as an *in vitro* model to delineate the effect of various lipid regulating agents on lipid metabolism. The effect of camphene on cholesterol biosynthesis was compared with the potent lipid lowering drug, mevinolin. Mevinolin is a commercially available statin, a member of the drug family systemically administered to hyperlipidemic individuals to inhibit HMG-CoA reductase and consequently lower the amounts of blood circulating total cholesterol, LDL-cholesterol and triglycerides [9], [10]. The concentration of mevinolin resulting in maximal inhibition of cholesterol biosynthesis is 37 µM in HepG2 cells [54]. We compared the effects of camphene and mevinolin on cholesterol biosynthesis in HepG2 cells. Cholesterol biosynthesis in the cells was stimulated by LPDS. After incubation for 1 h and 18 h with camphene (37 µM) and mevinolin (37 µM), lipids were extracted and total cholesterol was determined by gas chromatography. After 1 h, the cholesterol content was decreased by 20% (*p*<0.001) and 20.7% (*p*<0.01) in cells incubated with camphene and mevinolin, respectively, as compared to control cells exposed to LPDS alone (Figure 1). Further, incubation of HepG2 cells camphene and mevinolin for 18 h reduced cholesterol concentration by 10.4% (*p*<0.05) and 33.4% (*p*<0.001), respectively (Figure 1). It should be noted that the average cholesterol content was 4.774+0.883 µg/mg of total protein in control cells. In the 1 h incubation period, camphene exhibited a more dramatic effect than mevinolin, whereas after 18 h of incubation, the effect of mevinolin was increased, while camphene had no significant further effect.

### Camphene acts independently of HMG-CoA reductase activity

**Figure 2:**
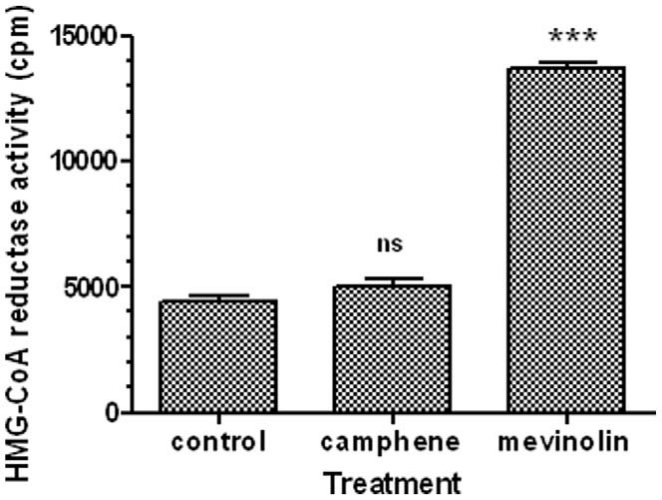
Effect of camphene and mevinolin on HMG-CoA reductase activity in HepG2 cells. On day 7, HepG2 cells were incubated with camphene (37 µM) and mevinolin (37 µM) for 18 h. After incubation, cell extracts were prepared and assayed for HMG-CoA reductase activity as described in the Methods section. HMG-CoA reductase activity is expressed in cpm. Results are from three independent experiments in triplicates and expressed as the mean ± SD. Significant differences between values were determined by the Student-Newmann-Keuls test. *p*<0.001 (***) and ns (non significant).

In order to investigate whether the camphene-induced anti-hyperlipidemic activity was dependent on HMG-CoA reductase, the enzyme activity was determined in HepG2 cells extracts. The effect of camphene on HMG-CoA reductase activity was also compared with the mevinolin-induced effect. Changes in HMG-CoA reductase activity were monitored by determining the incorporation of [^14^C]HMG-CoA into mevalonate. Cells were incubated for 18 h in the presence of camphene (37 µM) and mevinolin (37 µM), and cell extracts were prepared and assayed for HMG-CoA reductase activity as described in the Methods section. As shown in Figure 2, mevinolin treatment caused a 3-fold increase in HMG-CoA reductase activity as compared to untreated cells (*p*<0.001). In contrast, camphene treatment resulted in negligible changes in HMG-CoA reductase activity (115% of control, *p*>0.05). These results indicated that camphene exhibited a cholesterol-lowering activity via a process independent of inhibition of HMG-CoA reductase. Apparently, the cholesterol-lowering ability of camphene is mediated via a novel metabolic process distinctly different than the process of inhibition of HMG-CoA reductase induced by mevinolin.

### Effect of camphene on intracellular cholesterol ester content

**Figure 3:**
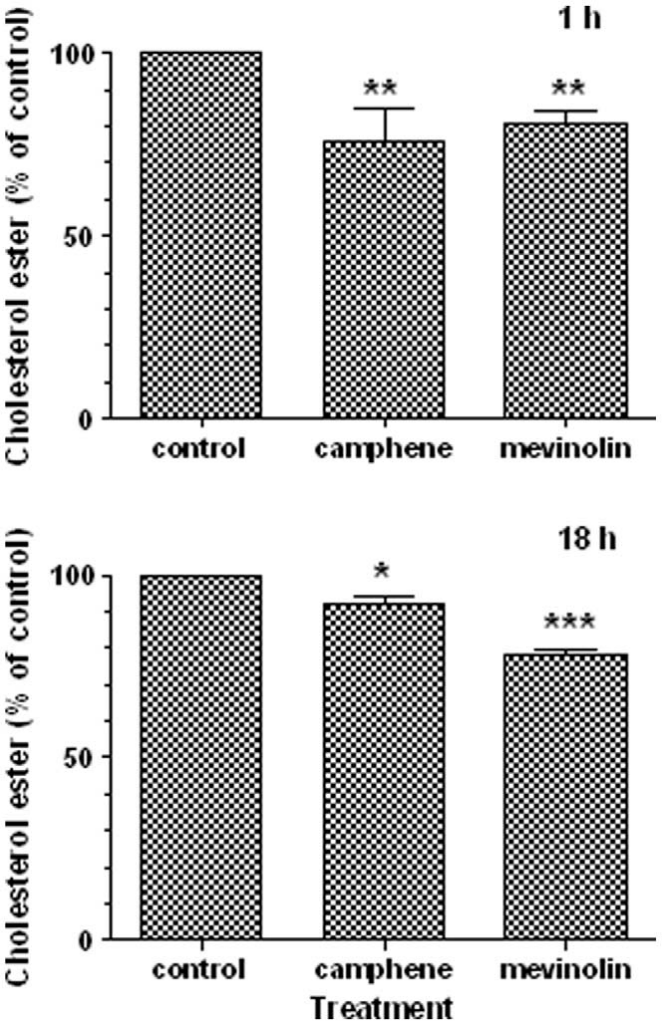
Effect of camphene and mevinolin on cholesterol ester contents in HepG2 cells. On day 7, HepG2 cells were incubated with camphene (37 µM) and mevinolin (37 µM) for 1 h and 18 h. Lipids were extracted and separated by TLC. Cholesterol ester contents were quantified by gas chromatography. Cholesterol ester concentrations in untreated cells (control) were defined as 100% and the levels in treated cells were plotted relative to that value. Results are from three independent experiments performed in triplicates and expressed as the mean ± SD. *p*<0.05 (*); *p*<0.01 (**); *p*<0.001 (***) by the Student-Newmann-Keuls test.

To further examine the effect of camphene, cholesterol ester levels were determined. HepG2 cells were incubated with camphene (37 µM) and mevinolin (37 µM) for a period of 1 h and 18 h. Subsequently, lipids were extracted and cholesterol esters were separated by TLC and measured by gas chromatography. Incubation of HepG2 cells with camphene significantly decreased cholesterol ester concentration by 24% (*p*<0.01) as compared to the control (Figure 3). As shown in this Figure, treatment for 1 h with camphene and mevinolin led to a significant decrease in the cholesterol ester concentration as compared to untreated cells. Treatment with camphene for 18 h significantly decreased intracellular cholesterol ester by 7.7% (*p*<0.01) compared to cholesterol ester levels in control cells (Figure 3). Incubation with mevinolin for 18 h resulted in a decrease of 22% (*p*<0.001). Mevinolin treatment had a different effect than the camphene-induced effect, implying that mevinolin may have higher bioavailability.

### Proliferation and protein content of treated HepG2 cells

We investigated whether the hypolipidemic effects of camphene and mevinolin were associated with changes in cell proliferation and protein content. Treatment of HepG2 cells with camphene and mevinolin for various times ranging from 4 h to 24 h showed no significant effect on the cell number as determined by the Trypan blue method (results not shown). Moreover, camphene was shown to suppress hyperlipidemia without affecting the global cell protein content in the *in vitro* HepG2 cell model (Table 6). Camphene was also confirmed to have no significant effect on cell proliferation as measured by the MTT cell growth assay method (Table 7), suggesting that camphene is no cytotoxic under the present experimental conditions.

**Table 6:** Effect of MGO and camphene on whole protein content of HepG2 cells.

	Protein (µg/µl)	
Treatment	1 h	18 h
control	7.714±0.957	8.150±0.887
camphene	7.816±1.197	8.266±1.028
mevinolin	7.548±0.956	8.615±1.257

On day 7 (see Methods), identical cultures of HepG2 cells were incubated with camphene (37 µM) and mevinolin (37 µM) for 1 h and 18 h, and subsequently whole cell protein was quantified. The measurements shown were obtained from three independent experiments performed in triplicate and expressed as the mean values ± SD. *p*>0.05 by the Student-Newmann-Keuls test.

**Table 7:** Cytotoxicity of camphene.

Treatment	MΤΤ (% of control)
control	100
camphene 25 µM	99.57±0.74
camphene 50 µM	98.87±2.15
camphene 100 µM	98.05±2.3

Confluent HepG2 cells were exposed to various concentrations of camphene (25, 50 and 100 µM) for 24 h and cytotoxicity was determined with the MTT assay (Methods section). The measurements shown were obtained from three independent experiments performed in triplicate and expressed as the mean values ± SD. *p*>0.05 by the Student-Newmann-Keuls test.

## Discussion

Disorders of lipid metabolism are the primary risk factor for cardiovascular disease. The known lipid-lowering drugs (statins, fibrates, bile acid sequestrants, etc) regulate the lipid metabolism by different mechanisms but also have many side effects in patients [55]–[57]. Therefore, various pharmacologically active molecules are under intensive investigation in order to find new therapies for dislipidemia [58], [59]. Lately, the protective effect of chios mastic gum against LDL oxidation was well documented as it was shown that triterpenes present in MG-derived essential oil exhibit antioxidant/antiatherogenic effect on LDL [30], [60]. Moreover, several studies using an aqueous extract from *P.lentiscus* leaves and MG powder have shown a strong hepatoprotective effect in rats and in humans [61], [62]. In addition, MG powder could have a cardioprotective role in humans without any substantial adverse effects in humans and animals [63], [64]. In the present study, the activity of MGO and camphene on plasma cholesterol and triglyceride levels was investigated in a rat model in which the circulating levels of total cholesterol, LDL-cholesterol and triglycerides were substantially increased by administration of the detergent, Triton WR-1339. This compound inhibits lipoprotein lipase resulting in increased cholesterol and triglycerides levels in plasma and thus it has been widely used for induction of acute hyperlipidemia [65] and for assay of putative hypocholesterolemic drugs [52], [66]–[68]. Initially, the effect of MGO was examined in naïve rats. In these animals, MGO drastically reduced plasma cholesterol and triglyceride levels (Table 1). Furthermore, a similar effect was observed when the animals were injected with MGO prior to induction of hyperlipidemia with Triton WR-1339 (Table 2). MG has already been associated with cardiovascular protection by inhibiting human LDL oxidation [30] and because of the presence of triterpenes causing an antioxidant/antiatherogenic effect [60]. Moreover, a beneficial action of MG powder was observed on serum total and LDL-cholesterol in healthy human subjects [62].

The chemical composition of MGO has been analysed but as yet no correlation has been made between the hypolipidemic activity with anyone of its individual constituents. In this study, we have shown for the first time that the hypolipidemic activity of MGO is associated with one of its constituents, namely camphene. Interestingly, camphene represents only a minor constituent (0.83%) of MGO [42]. Treatment with camphene alone revealed that cholesterol and triglyceride levels were inhibited less than with MGO administration (Tables 2 and 3). Moreover, a higher dose of camphene was required to achieve the same hypolipidemic effect as MGO (Table 5). This result was attributed to the potential synergistic action between camphene and other MGO component(s). However, when camphene was administered with each one of the major MGO individual components, no further decrease in cholesterol and triglyceride levels was observed (data not shown). On the other hand, the hypolidemic effect was restored to almost the same level obtained with MGO when camphene was used in combination with all the 5 major constituents of MGO (Table 4), implying a synergism among all these constituents. Camphene is a bicyclic monoterpene and a constituent of many essential oils derived from various plants [69]–[73]. It is used as a food additive for artificial flavoring as well as in the preparation of fragrances and in the manufacture of synthetic camphor and insecticides [74]. Recently, it was shown to have a protective effect against oxidative stress [75].

To gain some insights about the mechanism of action of camphene hypolipidemic activity, we investigated its effect on HMG-CoA reductase activity in HepG2 cells. It is well known that statins decrease total, free and esterified cholesterol concentrations in HepG2 cells [76], [77] by directly inhibiting HMG-CoA reductase. We showed that camphene did not significantly inhibited HMG-CoA reductase activity (Figure 2), implying that its hypolipidenic activity is *via* an unidentified yet mechanism different than that utilized by statins. Although, camphene treatment had no effect on HMG-CoA reductase activity, a decrease in free cholesterol and cholesterol ester levels was observed (Figures 1 and 3).

It should be noted that camphene is a monoterpene present in several essential oils derived from plants of diverse species. In plants, monoterpenes are products of the isoprenoid biosynthetic pathway. It was previously shown that only cyclic monoterpenes in which oxygen is a substituent have the capacity to inhibit hepatic HMG-CoA reductase activity, whereas pinene and camphene that contain no oxygen substituents had no significant effect [78]. It appears that the oxy- and hydroxy- substituted cyclic monoterpenes act in a similar manner as the oxy-substituents of cholesterol such as 25-hydroxy cholesterol that is far more potent inhibitor than cholesterol itself [79].

In our rat model, Triton WR-1339 acts as a surfactant and suppresses the action of lipases to block the uptake of lipoproteins from circulation by extra-hepatic tissues, resulting in increased blood lipid concentration [52], [65]. Therefore, the lipid-lowering effect caused by the administration of camphene may be due to reactivation of lipolytic enzymes for early clearance of lipids from circulation in Triton-induced hyperlipidemia. The observation that administration of MGO in naïve rats led to the same reduction in their plasma total cholesterol, LDL-cholesterol and triglyceride levels, implies that camphene may act as an LPL activator. Previous studies have shown that compounds that increase LPL activity decrease plasma cholesterol and triglyceride levels contributing to an antiatherogenic effect [80]. Further, the fact that camphene acts independent of HMG-CoA reductase would allow its use not only as a single agent, but also in combination with statins, the most widely prescribed class of drugs for lowering cholesterol according to the American Heart Association. A suggested combined treatment with camphene and a statin would allow the use of low doses of statin, which is known to occasionally result to liver injury and often in patient non-compliance. Alternatively, it would be possible to use camphene with plant-derived stanol and sterol esters, which are compounds that competitively inhibit the absorption of cholesterol and thus lower its level in plasma [81]–[83]. Camphene can be synthetically prepared from α-pinene (CAS No. 79-92-5: Screening information Data Set (SIDS) of OECD High Volume Chemicals Programme, 1993). Taken together the hypolipidemic activity of camphene and the readiness to manufacture this monoterpene, we propose that camphene could develop to an alternative hypolipidemic drug. In conclusion, further investigations are warranted for the development of camphene as a hypolipidemic agent.
